# Analysis of Differences in the Chemical Composition of Glycosides and Sugars between Four Forms of Fresh Rehmanniae Radix

**DOI:** 10.3390/molecules28247995

**Published:** 2023-12-07

**Authors:** Lu Xu, Xiaokai Guo, Shujuan Xue, Ruiyi Di, Suiqing Chen

**Affiliations:** 1 Henan Provincial Key Laboratory of Chinese Medicine Resources and Chinese Medicine Chemistry, Henan University of Chinese Medicine, Zhengzhou 450046, China; 15939131537@163.com (L.X.); scout1@126.com (X.G.); sjxue3901@163.com (S.X.); 17837167975@163.com (R.D.); 2 Collaborative Innovation Center of Research and Development on the Whole Industry Chain of Yu-Yao, Henan University of Chinese Medicine, Zhengzhou 450046, China

**Keywords:** Rehmanniae Radix, iridoid glycosides, sugars, chemical composition

## Abstract

Fresh Rehmanniae Radix, as well as its processed products, are widely used in the clinical practice of traditional Chinese medicine. It is mainly available in four forms: fresh Rehmanniae Radix, raw Rehmanniae Radix, prepared Rehmanniae Radix, and nine-steamed, nine-dried Rehmanniae Radix. Pharmacological studies have shown that all Rehmanniae Radix forms contain iridoid glycosides and sugar compounds with various effects, including hypoglycemic, anti-inflammatory, neuroprotective, immunological enhancement, and bone marrow hematopoiesis-promoting activities. Differences in the efficacy among these Rehmanniae Radix forms and their processed products have been attributed to variations in their chemical compositions, particularly in iridoid glycosides and sugar compounds; however, the specific compositional differences in glycosides and sugars among the four forms of Rehmanniae Radix have not been clarified. Therefore, this study aims to qualitatively characterize the iridoid glycosides and sugar compounds in fresh Rehmanniae Radix, raw Rehmanniae Radix, prepared Rehmanniae Radix, and nine-steamed, nine-dried Rehmanniae Radix.

## 1. Introduction

Rehmanniae Radix, also known as Rehmannia glutinosa Libosch, is a plant belonging to the family Scrophulariaceae. Its roots can be used in fresh or dried form. Rehmanniae Radix was first documented in the ancient Chinese text *Shennong Bencao Jing*, and it was classified as a superior herb [1]. The main components of Rehmanniae Radix include iridoid glycosides, phenylethanoid glycosides, anthraquinones, sugars, amino acids, volatile oils, and inorganic substances [2,3,4,5,6]. Our research group has previously studied the chemical composition of Rehmanniae Radix, and the results showed significant differences in the chemical composition among fresh Rehmanniae Radix (FRR), raw Rehmanniae Radix (RRR), prepared Rehmanniae Radix (PRR), and nine-steamed, nine-dried Rehmanniae Radix (NRR), particularly in iridoid glycosides and sugar compounds [7,8,9]. Furthermore, there is a certain correlation between the color of Rehmanniae Radix sections and the content of iridoid glycosides and oligosaccharides, with the section color deepening as the content of iridoid glycosides and oligosaccharides decreases and the content of monosaccharides and polysaccharides increases. This indicates that processing methods have a certain influence on the chemical composition of Rehmanniae Radix [10]. The efficacy of traditional Chinese medicine after oral administration is based on its inherent chemical components [11,12]. Pharmacological studies have shown that all Rehmanniae Radix forms contain iridoid glycosides and sugar compounds with various effects, including hypoglycemic, anti-inflammatory, neuroprotective, immunological enhancement, and bone marrow hematopoiesis-promoting activities [13,14,15,16,17,18,19,20,21]. Differences in efficacy among these Rehmanniae Radix forms and their processed products have been attributed to variations in their chemical compositions, particularly in iridoid glycosides and sugar compounds [22,23]. Studies have shown that iminosugars and sugar derivatives as antidiabetic agents, in combination with this study, hint at the importance of Rehmanniae’s antidiabetic properties, as well as their synthetic efforts toward the development of antidiabetic medicine [24,25]. The processing methods of the four forms of Rehmanniae Radix result in changes to their medicinal properties and efficacy. However, the chemical composition of Rehmanniae Radix undergoes either qualitative or quantitative changes after processing, which directly or indirectly affects its efficacy [26,27,28].

## 2. Results

**Figure 1:**
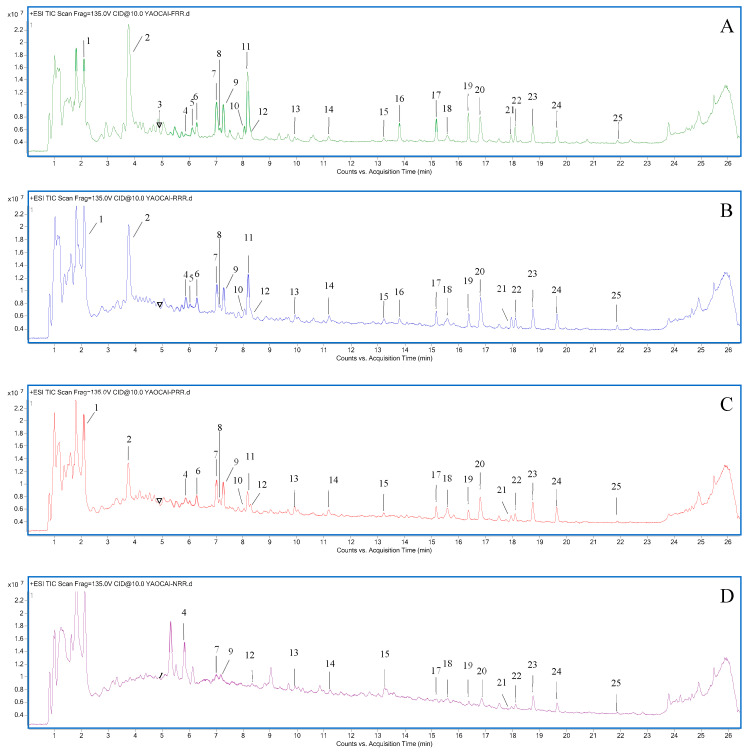
(**A**) TIC of iridoid glycosides in FRR, (**B**) TIC of iridoid glycosides in RRR, (**C**) TIC of iridoid glycosides in PRR, and (**D**) TIC of iridoid glycosides in NRR. (The Numbers represent the corresponding compounds are detailed in Supplementary Table S1).

**Figure 2:**
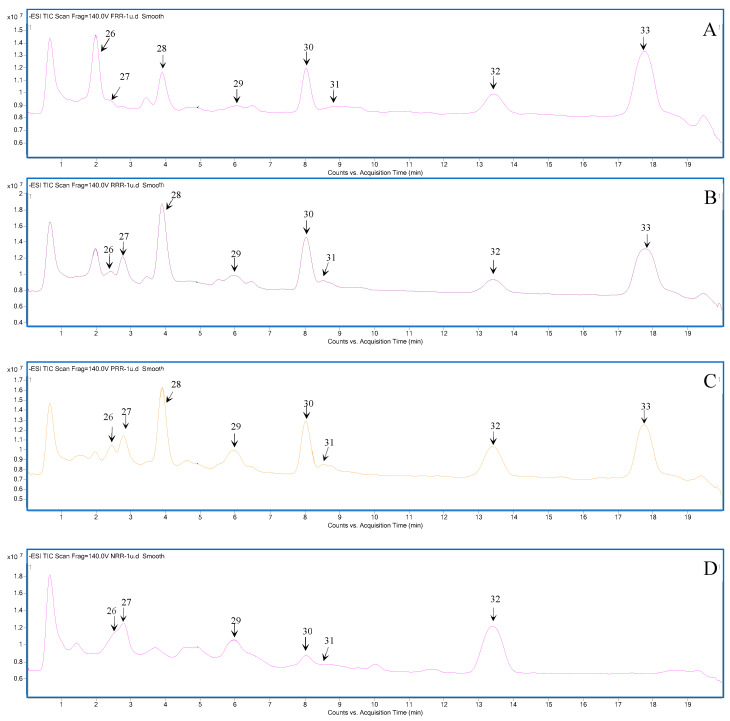
(**A**) TIC of sugars in FRR, (**B**) TIC of sugars in RRR, (**C**) TIC of sugars in PRR, and (**D**) TIC of sugars in NRR. (The Numbers represent the corresponding compounds are detailed in Supplementary Table S1).

**Figure 3:**
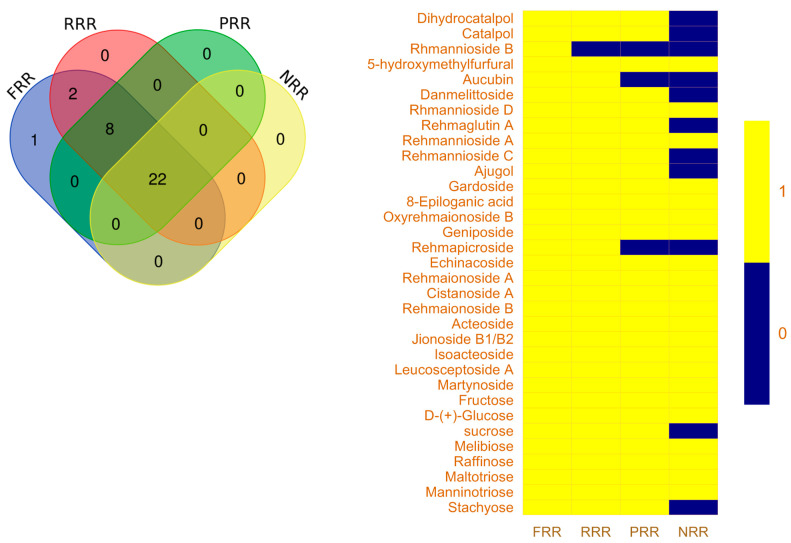
Chemical composition differences between FRR, RRR, PRR, and NRR.

The total ion chromatograms (TICs) of FRR, RRR, PRR, and NRR are shown in Figure 1 and Figure 2, and the chemical compositions are listed in Supplementary Table S1. Chemical composition differences are shown in Figure 3. The compounds of glycosides and sugars were more abundant in FRR than in the other three forms and, with increasing complexity of the processing technology, the types of these chemical components gradually reduced. The compounds were identified based on the fragmentation patterns of reference standards. For compounds without reference standards, MassHunter software 10.0 (Agilent) was used for molecular formula prediction. Structural identification of compounds with a mass error within 10 ppm was carried out based on the information about secondary ion fragments specific to that compound class.

### 2.1. Identification of Iridoid Glycoside Compounds

In our previous study [10], the identification of iridoid glycosides, as well as their glycoside compounds, was conducted. Compound **1** was speculated to be dihydrocatalpol; compound **3**, rehmannioside B; compound **10**, rehmannioside C; and compound **13**, 8-epiloganic acid. All these compounds were considered iridoid glycoside compounds.

Compound **12**: In positive ion mode, MassHunter software provided the most probable molecular formula for compound **12**, as 397.1104 (C_16_H_22_O_10_). Through referring to the literature [29], and analyzing the MS/MS spectrum of this compound, we inferred it to be gardoside.

### 2.2. Identification of Rehmaionoside Compounds

Compound **18**: In positive ion mode, an [M+Na]^+^ ion of compound **18** was observed at *m*/*z* 413.2149 (C_19_H_34_O_8_Na). The MS/MS spectrum exhibited an ion fragment at *m*/*z* 211.1692, indicating a loss of 180 Da corresponding to one glucose molecule. The peaks at *m*/*z* 193.1592 and 175.1484 indicated the consecutive loss of two water molecules, suggesting the presence of two hydroxyl groups. In referring to the literature, and considering the molecular weight and structural composition of this molecular formula, compound **18** was inferred to be a rehmaionoside compound.

Compound **20**: In positive ion mode, an [M+Na]^+^ ion of compound **20** was observed at *m*/*z* 413.2149 (C_19_H_34_O_8_Na), indicating its isomeric relationship with compound **18**. The MS/MS spectrum also exhibited ion fragments at *m*/*z* 211.1692, 193.1592, and 175.1484, indicating that compound **20** is also a rehmaionoside compound.

According to the literature [2], rehmaionoside A and rehmaionoside B are scutellarein compounds, and they are isomers. Therefore, compound **18** was tentatively identified as rehmaionoside A, and compound **20** as rehmaionoside B.

Compound **14**: In positive ion mode, an [M+Na]^+^ ion of compound **14** was observed at *m*/*z* 429.2097 (C_19_H_34_O_9_Na). The MS/MS spectrum showed an ion fragment at *m*/*z* 227.1642, indicating a loss of 180 Da corresponding to one glucose molecule. The peaks at *m*/*z* 209.1537 and 191.1430 indicated the consecutive loss of two water molecules, suggesting the presence of two hydroxyl groups. Based on the fragmentation pattern observed, which is similar to that of scutellarein A/B, compound **14** was inferred to be a scutellarein compound. Through referring to the literature, and considering the molecular weight and structural composition of this molecular formula, compound **14** was identified as oxyrehmaionoside B.

### 2.3. Identification of Phenylethanoid Glycoside Compounds

Based on previous laboratory identification, compounds **17**, **19**, and **23** were identified as phenylethanoid glycosides, namely echinacoside, cistanoside A, and isoacteoside, respectively. Compound **16** was identified as a Rehmannia glycoside compound, rehmapicroside.

Compounds **22**, **24**, and **25**: The most accurate molecular weights and probable molecular formulas obtained through MassHunter software were as follows: for compound **22**, [M+NH_4_]^+^ [30] had an *m*/*z* of 832.3241; for compound **24**, [M+Na]^+^ had an *m*/*z* of 661.2102; and for compound **25**, [M+Na]^+^ had an *m*/*z* of 675.2258. In the MS/MS spectra, the ion fragments of compounds **22**, **24**, and **25** were at *m*/*z* 195.0654, 179.0702, and 195.0652, respectively, suggesting that these compounds belong to the benzyl alcohol glycoside class. Referring to the literature [31], and based on the provided information, compound **22** was speculated to be jionoside B1/B2, compound **24** was believed to be leucosceptoside A, and compound **25** was presumed to be martynoside.

### 2.4. Identification of Rehmapicroside Compounds

Based on preliminary laboratory identification, compound **16** was classified as a cardenolide compound, specifically rehmapicroside.

### 2.5. Identification of Furanylaldehyde Compounds

Through the analyzation standard compounds, we determined that compound **4** is 5-hydroxymethylfurfural, with an [M+H]^+^ *m*/*z* of 127.0390. Further analysis of the MS/MS spectra revealed an ion fragment peak at *m*/*z* 109.0285, indicating a loss of 18 Da, which corresponds to the loss of one molecule of H_2_O.

### 2.6. Fragmentation Pattern Analysis

A total of 33 compounds were identified in Rehmanniae Radix, of which 27 had been previously identified through our research group. In this study, six additional compounds, namely compounds **12**, **14**, **20**, **22**, **24**, and **25**, were identified. For compounds **12**, **14**, **20**, **22**, **24**, and **25**, molecular formula prediction was initially conducted using MassHunter software. Molecular formulas with a mass error within ±10 ppm were selected, and structural identification and analysis of possible fragmentation patterns were carried out based on the secondary ion fragment information of these compounds, along with literature references.

**Figure 4:**
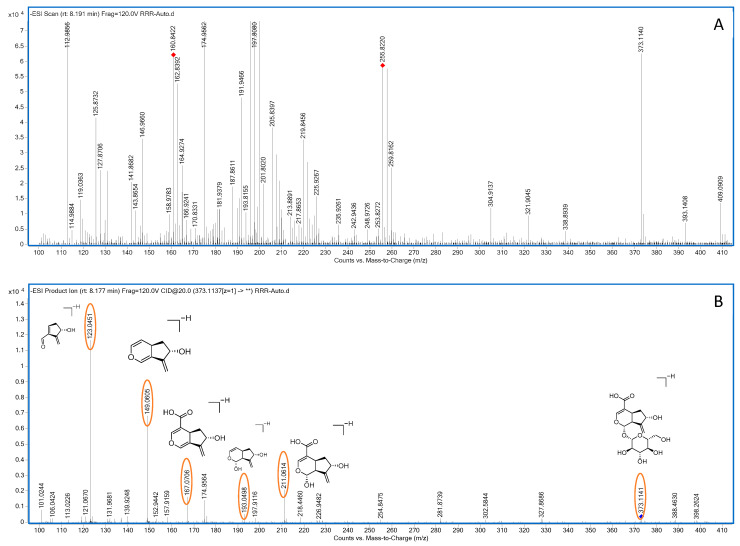
Negative-ion-mode ms1 spectrum (**A**) and two-dimensional ms2 spectrum (**B**) of gardoside (yellow circles are the ion markers of the illustrated fragment).

**Figure 5:**
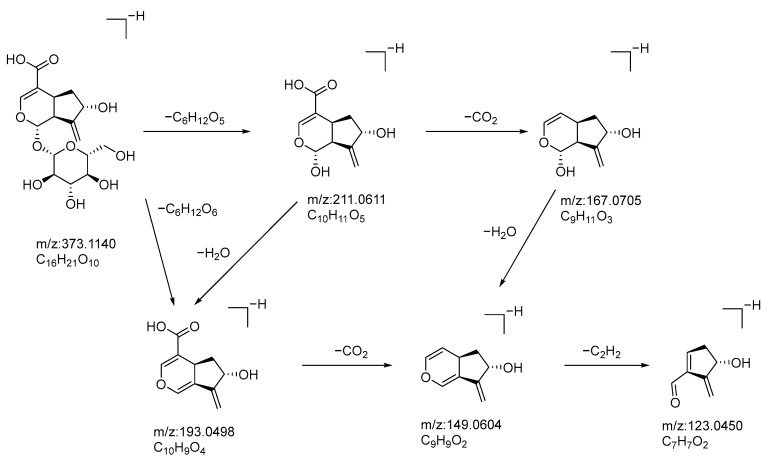
Possible fragmentation pathways of gardoside in the negative ion mode.

Compound **12**: In negative ion mode, in the ms1 spectrum, compound **12** showed a peak at *m*/*z* 373.1140 ([M−H]^−^), corresponding to C_16_H_21_O_10_, as shown in Figure 4. In the ms2 spectrum, the ion fragments had *m*/*z* values of 211.0614, 193.0498, 167.0705, 149.0604, and 123.0450, as seen in Figure 4. Among these, the ion fragment at *m*/*z* 211.0614 corresponded to the loss of C_6_H_10_O_5_ from *m*/*z* 373.1140 ([M−H]^−^), followed by the loss of one molecule of H_2_O, resulting in the ion fragment at *m*/*z* 193.0498 ([M−C_6_H_12_O_6_]). This suggests that the compound loses one molecule of glucose (180 Da), indicating the presence of one glucose molecule in the compound. The difference between *m*/*z* 193.0498 and *m*/*z* 149.0605 is 44 Da, suggesting a possible loss of CO_2_. The difference between *m*/*z* 149.0605 and *m*/*z* 123.0451 is 24 Da, indicating the possible loss of C_2_H_2_. Based on the observed ion fragments and referencing the literature [32], we inferred that the compound is likely to be gardoside. The possible fragmentation pathways of gardoside in negative ion mode can be seen in Figure 5.

**Figure 6:**
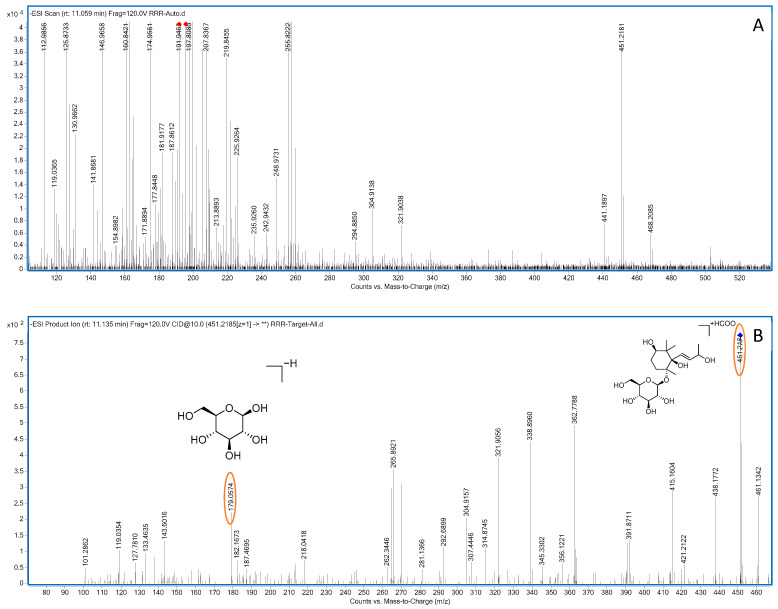
Negative-ion-mode ms1 spectrum (**A**) and ms2 spectrum (**B**) of oxyrehmaionoside B (yellow circles are the ion markers of the illustrated fragment).

**Figure 7:**
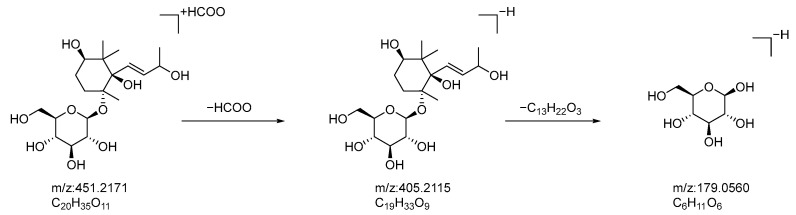
Potential fragmentation pathways of oxyrehmaionoside B in negative ion mode.

Compound **14**: In negative ion mode, in the ms1 spectrum, compound **14** exhibited an [M+HCOO]^−^ peak at *m*/*z* 451.2181 (C_20_H_35_O_11_), as shown in Figure 3. In the ms2 spectrum, the ion fragments had *m*/*z* values of 405.2115, 179.0560, and 119.0347, as observed in Figure 6. The ion fragment at *m*/*z* 405.2115 corresponded to the [M−H]^−^ peak, and the ion fragment at *m*/*z* 179.0560 indicated that the compound is associated with one molecule of glucose. Based on the molecular formula of the compound, the information from the ms2 spectrum, as well as referencing the literature [33], we hypothesized that the compound is oxyrehmaionoside B. The possible fragmentation pathway of oxyrehmaionoside B in negative ion mode can be seen in Figure 7.

**Figure 8:**
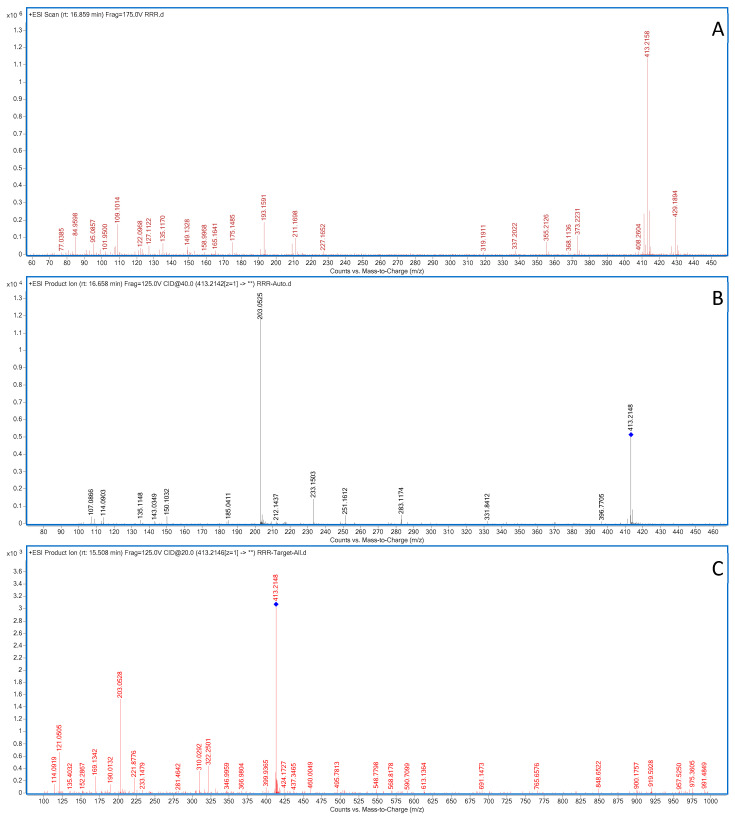
Positive-ion-mode ms1 (**A**) and ms2 (**B**) spectra of rehmannioside B, as well as the ms2 spectrum of rehmannioside A (**C**).

Compound **20**: In positive ion mode, the ms1 spectrum of compound **20** showed an [M+Na]^+^ peak at *m*/*z* 413.2158 (C_19_H_34_O_8_Na), indicating the same molecular weight as rehmaionoside A. In positive ion mode, rehmaionoside A and compound **20** were subjected to ms2 spectrometry analysis, as shown in Figure 8. It can be observed from the figure that the ms2 spectrum of compound **20** matches that of rehmaionoside A, suggesting that compound **20** is an isomer of rehmaionoside A. According to the literature [34], rehmaionoside A and rehmaionoside B are both iridoid glycosides and structural isomers. Therefore, compound **20** was tentatively identified as rehmaionoside B.

**Figure 9:**
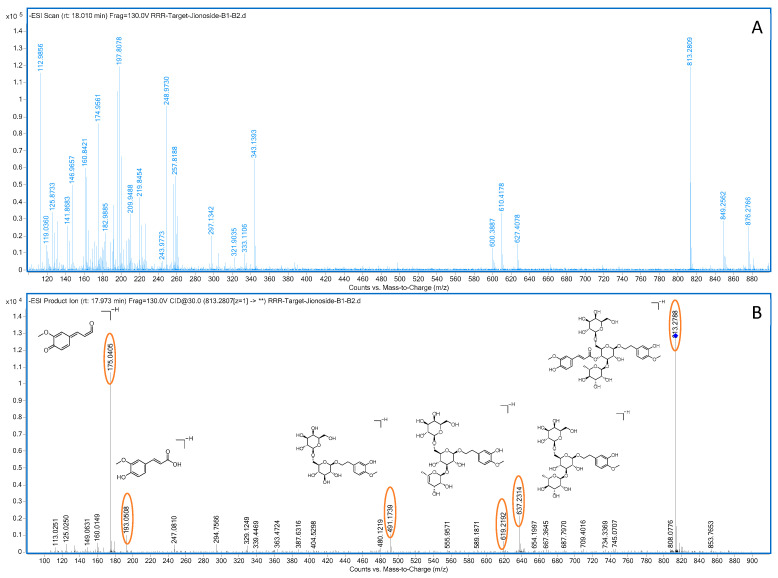
Negative-ion-mode ms1 spectrum (**A**) and ms2 spectrum (**B**) of jionoside B1/B2 (yellow circles are the ion markers of the illustrated fragment).

**Figure 10:**
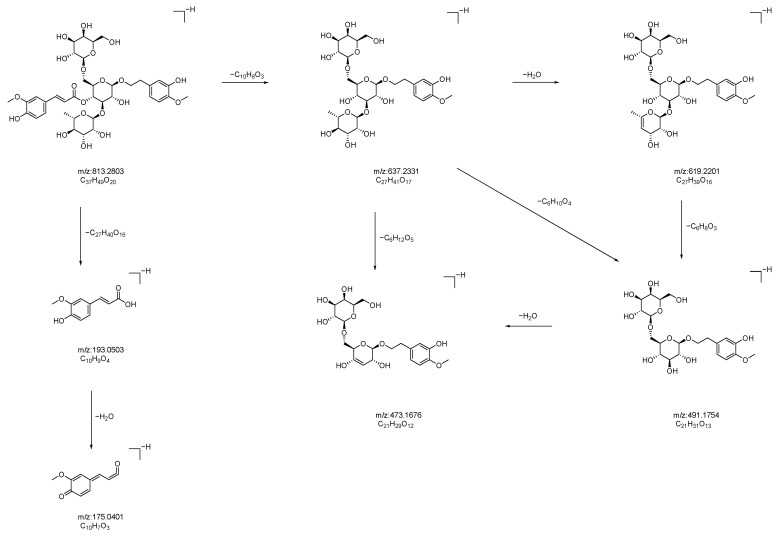
Possible fragmentation pathways of jionoside B1/B2 in negative ion mode.

Compound **22**: In negative ion mode, in the ms1 spectrum, compound **22** exhibited an [M−H]^−^ peak at *m*/*z* 813.2810 (C_37_H_49_O_20_), as shown in Figure 9. In the ms2 spectrum, the ion fragments had *m*/*z* values of 637.2331, 619.2201, 491.1754, 473.1676, 193.0503, and 175.0401, as observed in Figure 9. The ion fragment at *m*/*z* 637.2331 was formed via the loss of 176 Da from compound **22**, and an ion fragment at *m*/*z* 175.0402 was observed in the ms2 spectrum, suggesting the presence of one molecule of ferulic acid (C_10_H_8_O_3_) in the compound. Subsequently, the compound lost 164 Da, resulting in the appearance of the ion fragment at *m*/*z* 473.1676, indicating the presence of one molecule of syringic acid. Comparing the formed ion fragments from the ms2 spectrum with those reported in the literature [34,35,36], we believed that compound **22** is jionoside B1/B2 (isorhapontigenin glucoside B1/B2). The potential fragmentation pathway of jionoside B1/B2 in negative ion mode can be seen in Figure 10.

**Figure 11:**
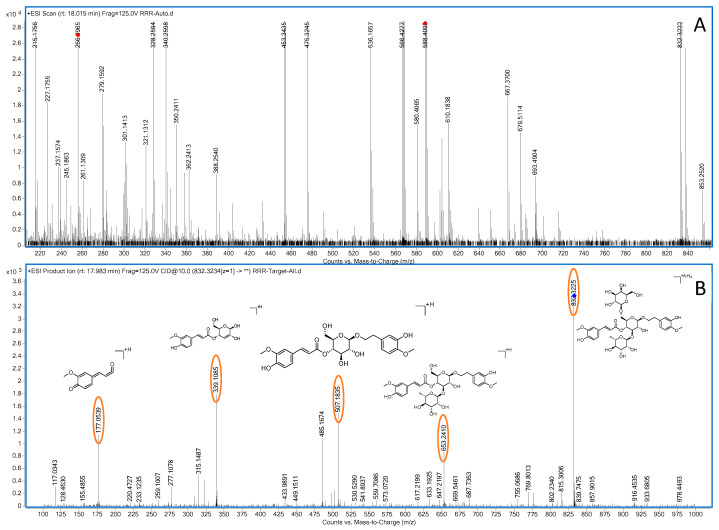
Positive-ion-mode ms1 spectrum (**A**) and ms2 spectrum (**B**) of catalpol-like iridoid glycosides B1/B2 (yellow circles are the ion markers of the illustrated fragment).

**Figure 12:**
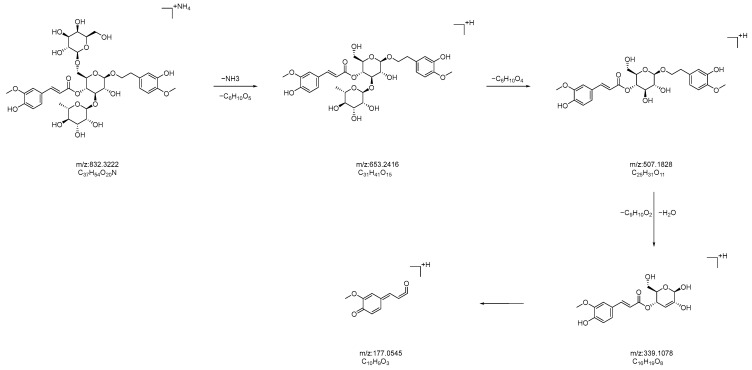
Potential fragmentation pathways of catalpol-like iridoid glycosides B1/B2 in positive ion mode.

In positive ion mode, in the ms1 spectrum, compound **22** showed an [M+NH_4_]^+^ peak at *m*/*z* 832.3222 (C_37_H_54_O_20_N), as depicted in Figure 11. Considering the negative ion mode, compound **22** was hypothetically identified as jionoside B1/B2 (isorhapontigenin glucoside B1/B2), and subjected to ms2 spectrometry analysis. The ion fragments in the ms2 spectrum had *m*/*z* values of 653.2416, 507.1828, 339.1078, and 177.0545. The ion fragment at *m*/*z* 653.2416 was formed via the loss of one molecule of C_6_H_10_O_5_ (162 Da) from jionoside B1/B2, followed by the loss of one molecule of C_6_H_10_O_4_ (146 Da), forming the ion fragment at *m*/*z* 507.1828. Subsequently, the ion fragment lost one molecule of C_9_H_10_O_2_ (150 Da), resulting in the appearance of the ion fragment at *m*/*z* 339.1078. Finally, the ion fragment at *m*/*z* 177.0545 was formed via the loss of one molecule of C_6_H_10_O_5_ (162 Da). The formed ion fragments in the ms2 spectrum are consistent with those reported in the literature [1,36]. The possible fragmentation pathways in positive ion mode are shown in Figure 12.

In conclusion, based on the aforementioned evidence, compound **22** is believed to be jionoside B1/B2, a phenylethanoid glycoside compound.

**Figure 13:**
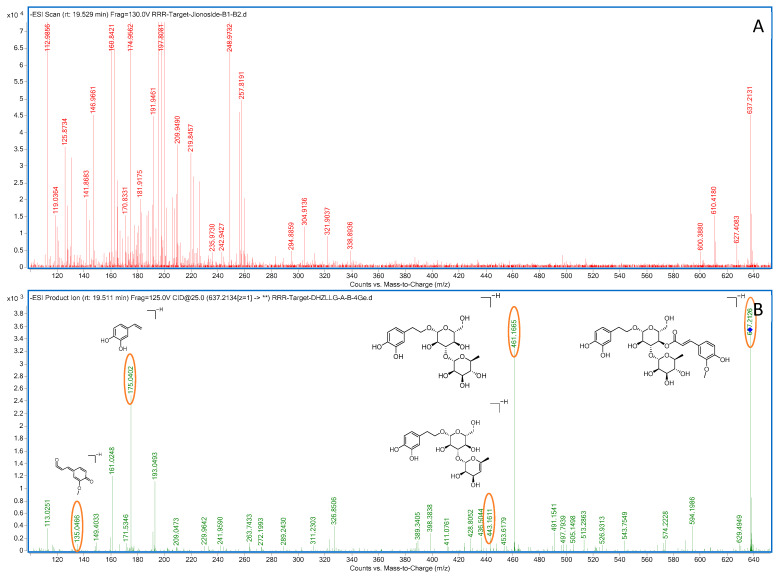
Negative-ion-mode ms1 spectrum (**A**) and ms2 spectrum (**B**) of white fruit saponin A. Yellow circles are the ion markers of the illustrated fragment.

**Figure 14:**
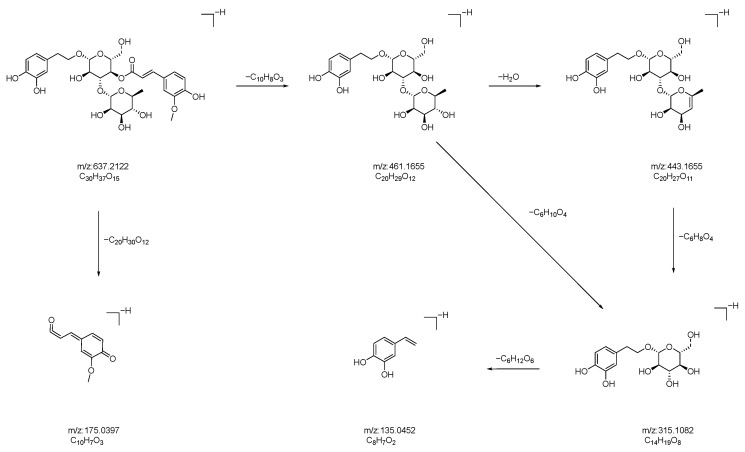
Possible fragmentation pathways of white fruit saponin A in negative ion mode.

Compound **24**: In negative ion mode, in the ms1 spectrum, compound **24** exhibited an [M-H]^−^ peak at *m*/*z* 637.2132 (C_30_H_37_O_15_), as shown in Figure 13. The ion fragments in the ms2 spectrum had *m*/*z* values of 461.1655, 443.1560, 315.1082, 175.0397, and 135.0452, as observed in Figure 13. The ion fragment at *m*/*z* 461.1655 corresponded to the loss of 176 Da from compound **24**, and the appearance of the ion fragment at *m*/*z* 175.0397 in the ms2 spectrum suggested the presence of a feruloyl moiety (C_10_H_8_O_3_) in this compound. Subsequently, the loss of 146 Da (C_6_H_10_O_4_) led to the formation of the ion fragment at *m*/*z* 315.1082. Lastly, the loss of 180 Da resulted in the ion fragment at *m*/*z* 135.0452, indicating the presence of a glucose moiety (C_6_H_12_O_6_) in this compound. Comparing the generated ion fragments with the reported data in [34,35], we inferred that this compound is leucosceptoside A, a benzyl alcohol glycoside compound. The possible fragmentation pathway in negative ion mode is shown in Figure 14.

**Figure 15:**
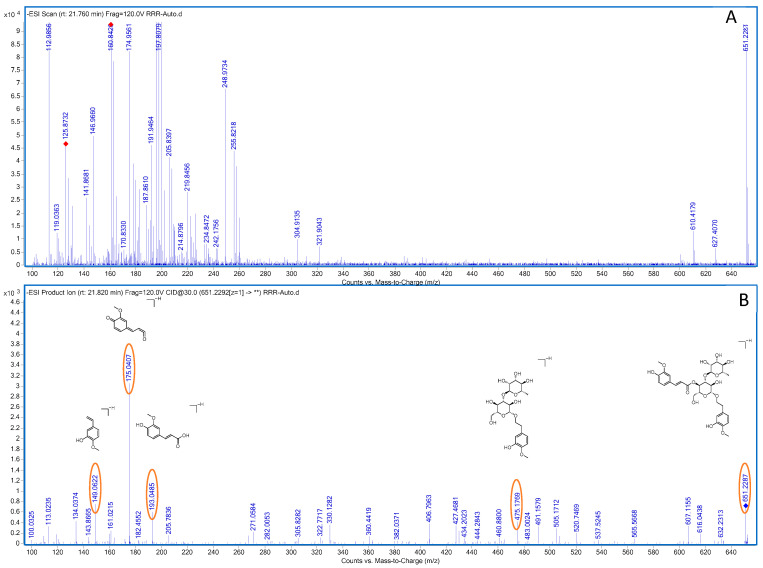
Negative-ion-mode ms1 spectrum (**A**) and ms2 spectrum (**B**) of rehmannoside. Yellow circles are the ion markers of the illustrated fragment.

**Figure 16:**
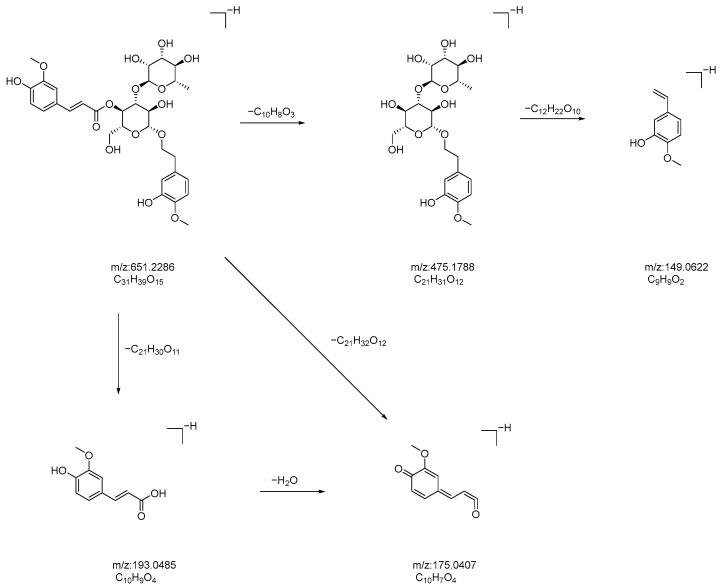
Potential fragmentation pathways of rehmannoside in negative ion mode.

Compound **25**: In negative ion mode, in the ms1 spectrum, compound **25** exhibited an [M-H]^−^ peak at *m*/*z* 651.2286 (C_31_H_39_O_15_), as shown in Figure 15. The ion fragments in the ms2 spectrum had *m*/*z* values of 475.1788, 193.0504, 175.0402, and 149.0622, as observed in Figure 15. The ion fragment at *m*/*z* 475.1788 corresponded to the loss of 176 Da from compound **25**, and the appearance of the ion fragment at *m*/*z* 175.0402 in the ms2 spectrum suggested the presence of a feruloyl moiety (C_10_H_8_O_3_) in this compound. The ion fragment at *m*/*z* 149.0622 in the ms2 spectrum corresponded to the loss of 302 Da from *m*/*z* 475.1788, which indicates the presence of a glucose moiety and a dehydrated rhamnose moiety (C_12_H_22_O_10_) in this compound. Comparing the generated ion fragments with the information in [34], we concluded that this compound is martynoside, a benzyl alcohol glycoside compound. The possible fragmentation pathway in negative ion mode is shown in Figure 16.

### 2.7. Selection of the Mobile Phase

During mass spectrometry analysis, we used acetonitrile-water, as well as acetonitrile-0.1% formic acid water, as solvent systems for the analysis of cyclic enol ether triterpenoids and their glycosides, respectively. The results showed that when using acetonitrile-0.1% formic acid water, better separation of components was achieved, and a higher response in mass spectrometry was obtained compared to acetonitrile-water. For the analysis of sugar components, we used 0.1% ammonia water-acetonitrile-0.1% ammonia water, various compositions of 30% acetonitrile water-80% acetonitrile water containing 0.1% ammonia water, 8 mM of ammonium formate water (pH = 9.8), and 8 mM of ammonium formate-acetonitrile (pH = 9.8). The results demonstrated that using 0.1% ammonia water-acetonitrile-0.1% ammonia water provided better separation of components, as well as a higher response in mass spectrometry. Consequently, 0.1% ammonia water-acetonitrile-0.1% ammonia water was chosen for the analysis of sugar components.

## 3. Discussion

The effectiveness of Rehmannia glutinosa (known as “dihuang” in Chinese) in the body is based on its chemical components. In this study, the chemical compositions of FRR, RRR, PRR, and PRR were characterized using UPLC-Q-TOF/MS technology. A total of 33 compounds were identified in dihuang in positive and negative ion modes, with 27 compounds previously identified through the research team, and six new compounds being identified in this study. The newly identified compounds include gardenoside, oxyrehmaionoside B, catalpol, jionoside B1/B2, betulinic acid glycoside A, and cataloside. The chemical composition of Rehmannia gradually decreased during the processing from FRR to NRR.

In FRR, a total of 33 compounds were identified in positive and negative ion modes, including 13 iridoid glycosides and their derivatives, eight sugars, seven phenylethanol glycosides, three violet ketones, one catalpol, and one furan aldehyde. RRR contained 32 identified compounds in positive and negative ion modes, including 12 iridoid glycosides and their derivatives, eight sugars, seven phenylethanol glycosides, three violet ketones, one catalpol, and one furan aldehyde. PRR had 30 identified compounds in positive and negative ion modes, including 11 iridoid glycosides and their derivatives, eight sugars, seven phenylethanol glycosides, three violet ketones, and one furan aldehyde. NRR had 22 identified compounds in positive and negative ion modes, including five iridoid glycosides and their derivatives, six sugars, seven phenylethanol glycosides, three violet ketones, and one furan aldehyde.

Our previous research focused on the study of cyclic enol ether triterpenoids in Rehmanniae Radix. It was found that the content of these compounds gradually decreased during the processing of Rehmanniae Radix and preparation of its different forms. Through this study, we observed that the highest number of compounds whose content reduced during the steaming and sun-drying processing of RRR to NRR is eight. These compounds include dihydrocatalpol, catalpol, aucubin, catalpol, catalpol, aucubin, saccharose, and isomaltose. Most of these compounds belong to cyclic enol ether triterpenoid monosaccharides, disaccharides, or sugar compounds. This phenomenon is related to the thermal instability of cyclic enol ether glycosides. During the processing of FRR to RRR, and then the subsequent production of PRR, followed by further processing to produce NRR, the high temperature caused the glycosidic bonds of cyclic enol ether glycosides to undergo hydrolysis, resulting in the cleavage and opening of the cyclic enol ether structure and ester bonds, leading to a decrease in their content.

The primary therapeutic properties of RRR are clearing heat, cooling blood, nourishing yin, and generating fluid. After processing, the main therapeutic properties shift to nourishing yin, replenishing blood, invigorating the essence, and filling the marrow. This change is related to the variation in the content of cyclic enol ether triterpenoids in Rehmanniae Radix after the processing and proportional changes between these compounds. During the steaming and sun-drying processing of Rehmanniae Radix to produce NRR, sucrose and isomaltose also undergo hydrolysis due to the influence of the high temperature and water. This indicates the importance of temperature control during production and processing.

In our previous research, the fragmentation patterns of cyclic enol ether glycosides and sugar components were analyzed, yielding the following results: In positive ion mode, monosaccharide glycosides of cyclic enol ethers undergo successive characteristic ion losses of 18 Da (H_2_O), 18 Da (H_2_O), and 126 Da (C_6_H_6_O_3_) to remove one glucose moiety. For cyclic enol ether glycosides containing two or more sugars, the same characteristic ion losses are observed successively until all sugar residues on the aglycone are removed. Subsequently, the aglycone undergoes fragmentation, primarily occurring at the cyclopentane ring, resulting in the typical characteristic ion loss of CO (28 Da) or CH_2_O (30 Da). In negative ion mode, monosaccharide glycosides of cyclic enol ethers directly lose one glucose moiety to generate a characteristic ion loss of C_6_H_12_O_6_ (180 Da). Cyclic enol ether glycosides containing two or more sugars continue to lose glucose moieties until all sugar residues on the aglycone are eliminated. Subsequently, the aglycone undergoes fragmentation, primarily at the carbon–carbon bond of the cyclopentane ring. Additionally, for cyclic enol ether glycosides with substituents at the 4-position of the aglycone, after the removal of glucose, the aglycone first loses the substituents at the 4-position before undergoing fragmentation of the cyclopentane carbon–carbon bond.

Regarding fragmentation patterns of sugar components, pentoses (five-carbon sugars) exhibited an [M−H]^−^ quasi-molecular ion peak at *m*/*z* 149.0455, followed by the loss of one water molecule to break the sugar ring, resulting in characteristic ions at *m*/*z* 131.0348, 113.0243, 85.0295, and 71.0138. The fragmentation pattern of hexoses (six-carbon sugars) was similar to that of pentoses, starting with an [M−H]^−^ quasi-molecular ion peak at *m*/*z* 179.0560, followed by the loss of one water molecule to generate characteristic ions at *m*/*z* 161.0453, 143.0347, 113.0243, 89.0244, and 71.0138. For disaccharides, an [M−H]^−^ quasi-molecular ion peak at *m*/*z* 341.1084 was observed. One of the two hexose units consecutively lost water molecules, followed by the cleavage of the sugar chain, ultimately resulting in the loss of one hexose unit to form a monosaccharide characteristic ion at *m*/*z* 179.0560. Subsequent loss of one water molecule generated characteristic ions at *m*/*z* 161.0453, 143.0347, 113.0243, 89.0244, and 71.0138. The fragmentation pattern of trisaccharides showed an [M-H]^−^ quasi-molecular ion peak at *m*/*z* 503.1612. The first monosaccharide unit sequentially lost water molecules, broke the sugar chain, and eliminated one hexose unit to form a disaccharide characteristic ion at *m*/*z* 341.1081. The disaccharide then continued to lose one hexose unit, resulting in a monosaccharide characteristic ion at *m*/*z* 179.

## 4. Materials and Methods

### 4.1. Instruments, Chemicals, and Reagents

The Agilent 6546LC/Q-TOF system was used for the experiments, and was equipped with an online degasser, a binary pump, a diode array UV detector (DAD), and a temperature-controlled autosampler. The system was operated with MassHunter software, including Mass Profiler 10.0 and Mass Profiler Professional 15.1 data processing software. A rotary evaporator (NVC2200, EYELA, Tokyo, Japan), as well as a high-speed centrifuge (IW-3021HR, Anhui Jiawen Instrument Equipment Co., Ltd., Anhui, China), were also used. Chromatographic-grade acetonitrile, formic acid (LC/MS grade, 98% purity, code: 147932500, ACROS), and ammonia solution (LC/MS grade, 98% purity) were purchased from Fisher Scientiifc (Bridgewater, NJ, USA). Ultrapure water was produced with a Milli-Q water system. Catalpol, 5-hydroxymethylfurfural, aucubin, danmelittoside, rehmannioside D, rehmannioside A, ajugol, geniposide, acteoside, fructose, D-(+)-glucose, sucrose, melibiose, raffinose, maltotriose, manninotriose, and stachyose (reference substance) were purchased from Beijing Zhongke Quality Inspection Biotechnology Co., Ltd., Beijing, China (batch numbers: Z-005-180315, Wkg17061502, T-080-170310, D-078-171216, 81720-08-3, D-083-170310, Y-220-170206, J-226-170206, M-086-180211, G-056-171216, P-050-171216, Z-038-171216, B20866, M-058-171216, 100274-201102, L01JY7030, and A-012-170426, respectively).

### 4.2. Preparation of Extracts and Polysaccharide Samples

Fresh Rehmanniae Radix (FRR) was collected from Wen County, Jiaozuo City, Henan Province, China. Raw Rehmanniae Radix (RRR) and prepared Rehmanniae Radix (PRR) were purchased from Henan Bailiao Huai Medicinal Science and Technology Development Co., Ltd., and nine-steamed, nine-dried Rehmanniae Radix (NRR) was purchased from Wen County Dapeng Huai Medicine Factory. All samples were identified as Rehmannia glutinosa (Gaert.) Libosch. ex Fisch. et Mey., by Professor Chen Suiqing of the Henan University of Chinese Medicine. We received approval for sampling in line with the regulations of project 81973477. The voucher specimen was deposited in a warehouse at the Henan Provincial Key Laboratory of Chinese Medicine Resources and Chinese Medicine Chemistry.

The extraction of FRR, RRR, PRR, and NRR solutions was conducted according to the extraction method for traditional Chinese medicine decoction [32]. Based on the extraction yields of each herb, 766 g of freeze-dried slices of FRR were soaked in 8-fold distilled water for 30 min, followed by reflux extraction for 40 min. The extract was filtered, and the residue was subjected to reflux extraction with 6-fold distilled water for an additional 30 min. The two filtrates were combined and concentrated to 900 mL using a rotary evaporator, resulting in a concentration of 0.75 g/mL for the FRR extract. Similarly, 832 g of RRR, 595 g of PRR, and 536 g of NRR were subjected to extraction following the same method. The combined filtrates were then vacuum-concentrated to 450 mL, obtaining concentrated extracts of fresh Rehmanniae Radix, raw Rehmanniae Radix, prepared Rehmanniae Radix, and nine-steamed, nine-dried Rehmanniae Radix, all at a concentration of 0.75 g/mL.

Appropriate volumes of the extracts of fresh Rehmanniae Radix, raw Rehmanniae Radix, prepared Rehmanniae Radix, and nine-steamed, nine-dried Rehmanniae Radix were diluted with water. After centrifugation at 12,000 rpm and 4 °C for 15 min, the supernatants were filtered through a 0.22 μm microporous membrane. UPLC-MS analysis was performed to analyze the iridoids and their glycosides, as well as the sugar components in the extracts.

### 4.3. Analytical Conditions

#### 4.3.1. Analysis of Cyclohexenone Terpenoids and Their Glycosides

(1) Chromatographic Conditions

The chromatographic column used was an ACQUITY UPLC HSS T3 column (2.1 × 100 mm, 1.8 μm). The mobile phase consisted of acetonitrile (B) and 0.1% formic acid in water (A). The gradient elution program was as follows: 0–1 min, 1% B; 1–3 min, 1–3% B; 3–10 min, 3–11% B; 10–22 min, 11–30% B; 22–25 min, 30–100% B; and 25–27 min, 100% B. The flow rate was set at 0.3 mL/min, with an injection volume of 3 μL. Detection was performed at a wavelength of 203 nm, and the column temperature was maintained at 30 °C.

(2) Mass Spectrometric Conditions

Dual atmospheric pressure chemical ionization (Dual AJS ESI) was used with a scanning range of 50–1000 Da in positive ion mode. The drying gas temperature (Gas Temp) was set at 325 °C, and the drying gas flow rate (Drying Gas) at 11 L/min. The nebulizer pressure (Nebulizer) was set at 40 psi, and the sheath gas temperature (Sheath Gas Temp) at 375 °C. The sheath drying flow (Sheath Drying Flow) was set at 10 L/min. The capillary voltage (VCap) was set at 4000 V, the nozzle voltage (Nozzle Voltage) at 0 V, the fragmentor voltage (Fragmentor) at 135 V, the skimmer voltage at 65 V, the Oct 1 RF Vpp at 750 V, and the collision energy at 10 V in positive ion mode. In negative ion mode, the capillary voltage was set at 3500 V, and the nozzle voltage at 1000 V, with all other parameters kept the same as in positive ion mode.

#### 4.3.2. Sugar Components

(1) Chromatographic Conditions

The chromatographic column used was a Waters BEH Amide column (2.1 × 100 mm, 1.7 μm), with a mobile phase of 0.1% ammonium hydroxide in acetonitrile (B) to 0.1% ammonium hydroxide (A). The following gradient elution program was applied: 0–17 min, 75% B; 17–18 min, 75–100% B; and 18–20 min, 100% B. The flow rate was set at 0.3 mL/min, the injection volume was 2 μL, and the column temperature was maintained at 35 °C.

(2) Mass Spectrometric Conditions

The dual electrospray ionization source (Dual AJS ESI source) was used with a scan range of 50–1000 Da in negative ion mode. The drying gas temperature was set at 300 °C with a flow rate of 10 L/min. The nebulizer pressure was 40 psi, the sheath gas temperature was 380 °C with a flow rate of 11 L/min, the capillary voltage was 3500 V, the nozzle voltage was 1000 V, the fragmentor voltage was 175 V, the skimmer voltage was 65 V, the Oct 1 RF Vpp was 750 V, and the collision energy was 25 V.

## 5. Conclusions

This study elucidated the compositional differences in iridoid glycosides and sugar compounds among four forms of Rehmanniae Radix, namely, fresh Rehmanniae Radix, raw Rehmanniae Radix, prepared Rehmanniae Radix, and nine-steamed, nine-dried Rehmanniae Radix. These findings provide a scientific basis for understanding the active constituents responsible for the therapeutic effects of Rehmannia in the body. Furthermore, they highlight the importance of temperature control during the processing of Rehmanniae Radix.

## Data Availability

The data presented in this study are available in the Supplementary Materials.

## Supplementary Materials

The following supporting information can be downloaded at: https://www.mdpi.com/article/10.3390/molecules28247995/s1, Supplementary Table S1: presents the chemical composition analysis of fresh Rehmanniae Radix(FRR), raw Rehmanniae Radix(RRR), prepared Rehmanniae Radix(PRR), and nine steamed and nine dried Rehmanniae Radix(NRR).
