# Supplementary material for: Analysis of Differences in the Chemical Composition of Glycosides and Sugars between Four Forms of Fresh Rehmanniae Radix
Source: Molecules. 2023 Dec 7;28(24):7995. doi: 10.3390/molecules28247995 (PMC10746076; doi:10.3390/molecules28247995)
Supplement: Supplementary file 1 [file molecules-28-07995-s001.zip › molecules-2680334-supplementary.pdf]

Analysis of Differences in the Chemical Composition of Glycosides and Sugars between Four Forms of Fresh Rehmanniae Radix

Lu Xu, Xiaokai Guo, Shujuan Xue, Ruiyi Di, Suiqing Chen*

Henan University of Chinese Medicine, Henan Provincial Key Laboratory of Chinese Medicine Resources and Chinese Medicine Chemistry, Zhengzhou Henan 450046, China

Table S1. presents the chemical composition analysis of fresh Rehmanniae Radix(FRR), raw Rehmanniae Radix(RRR), prepared Rehmanniae Radix(PRR), and nine steamed and nine dried Rehmanniae Radix(NRR).

No.	chemical name	RT/min	m/z ESI+	Error (ppm)	MS/MS	M+	m/z ESI-	Error (ppm)	MS/MS	M-	Formula	FRR	RRR	PRR	NRR
1	Dihydrocatalpol	2	387.1261	-0.17	185.0807 $[M-C_6H_{12}O_6]^+$<br>167.0702 $[M-C_6H_{12}O_6-H_2O]^+$	$[M+Na]^+$	363.1294	-0.17	201.0768 $[M-C_6H_{10}O_5]$-<br>183.0665 $[M-C_6H_{12}O_6]$-	$[M-H]^-$	$C_{15}H_{24}O_{10}$	√	√	√	-
2*	Catalpol	3.75	385.1106	0.19	183.0651 $[M-C_6H_{12}O_6]^+$<br>165.0546 $[M-C_6H_{12}O_6-H_2O]^+$	$[M+Na]^+$	407.1203	0.19	199.0615 $[M-C_6H_{10}O_5]$-<br>181.0505 $[M-C_6H_{10}O_5-H_2O]^-$	$[M+COOH]^-$	$C_{15}H_{22}O_{10}$	√	√	√	-
3	Rhmannioside B	4.808	547.1634	0.15	507.1705 $[M-H_2O]^+$<br>345.1177 $[M-C_6H_{10}O_5]^+$	$[M+Na]^+$	-	0.15	-	-	$C_{21}H_{32}O_{15}$	√	-	-	-
4*	5-hydroxymethylfurfural	5.806	127.039	0.23	109.0284 $[M-H_2O]^+$	$[M+H]^+$	125.0244	0.23	-	$[M-H]^-$	$C_6H_6O_3$	√	√	√	√
5*	Aucubin	6.07	369.1155	-0.06	167.0700 $[M-C_6H_{12}O_6]^+$<br>149.0596 $[M-C_6H_{12}O_6-H_2O]^+$	$[M+Na]^+$	391.1248	-0.06	183.0665 $[M-C_6H_{10}O_5]^-$<br>165.0194 $[M-C_6H_{12}O_6]^-$	$[M+COOH]^-$	$C_{15}H_{22}O_9$	√	√	-	-
6*	Danmelittoside	6.27	385.1106	-0.37	327.1069 $[M-2H_2O]^+$<br>165.0546 $[M-C_6H_{12}O_6-H_2O]^+$	$[M+Na]^+$	-	-0.37	-	-	$C_{15}H_{22}O_{10}$	√	√	√	-
7*	Rhmannioside D	7.01	709.2164	0.13	489.1594 $[M-C_6H_{12}O_6]^+$<br>363.0939 $[M-C_6H_{12}O_6-C_6H_6O_3]^+$	$[M+Na]^+$	731.2262	0.13	-	$[M+COOH]^-$	$C_{27}H_{42}O_{20}$	√	√	√	√
8	Rehmaglutin A	7.185	220.1181	-1.01	184.0969 $[M-H_2O]^+$<br>90.0550 $[M-C_6H_{10}O_3]^+$	$[M+NH_4]^+$	-	-1.01	-	-	$C_9H_{14}O_5$	√	√	√	-

Table S1. Cont

No.	chemical name	RT/ min	m/z ESI+	Error (ppm)	MS/MS	M+	m/z ESI--	Error (ppm)	MS/MS	M-	Formula	FRR	RRR	PRR	NRR
9*	Rehmannioside A	7.27	547.1636	0.07	345.1176 $[M-C_6H_{12}O_6]^+$<br>183.0653 $[M-2C_6H_{12}O_6]^+$	$[M+Na]^+$	569.1718	0.07	343.1033$[M-C_6H_{12}O_6]^-$<br>181.0503 $[M-C_6H_{12}O_6-C_6H_{10}O_5]^-$	$[M+COOH]^-$	$C_{21}H_{32}O_{15}$	√	√	√	√
10	Rehmannioside C	8.03	533.1842	0.12	331.1391 $[M-C_6H_{12}O_6]^+$<br>169.0859$[M-C_6H_{12}O_6-C_6H_{10}O_5]^+$	$[M+Na]^+$	509.1876	0.12	-	$[M-H]^-$	$C_{21}H_{34}O_{14}$	√	√	√	-
11*	Ajugol	8.16	371.1318	1.57	169.0860 $[M-C_6H_{12}O_6]^+$<br>151.0754 $[M-C_6H_{12}O_6-H_2O]^+$	$[M+Na]^+$	393.1411	1.57	167.0714 $[M-C_6H_{12}O_6]^-$<br>149.0606 $[M-C_6H_{12}O_6-H_2O]^-$	$[M+COOH]^-$	$C_{15}H_{24}O_9$	√	√	√	-
12	Gardoside	8.299	397.1104	-0.46	-	$[M+Na]^+$	373.114	-0.46	211.0620 $[M-C_6H_{10}O5]^-$<br>123.0457 $[M-C_6H_{12}O_6-C3H4O3]^-$	$[M-H]^-$	$C_{16}H_{22}O_{10}$	√	√	√	√
13	8-Epiloganic acid	9.9	399.1261	-0.08	197.0808 $[M-C_6H_{12}O_6]^+$<br>179.0703 $[M-C_6H_{12}O_6-H_2O]^+$	$[M+Na]^+$	375.1298	-0.08	213.0769 $[M-C_6H_{10}O_5]^-$<br>169.0870 $[M-C_6H_{10}O_5-CO_2]^-$	$[M-H]^-$	$C_{16}H_{24}O_{10}$	√	√	√	√
14	Oxyrehmaionoside B	11.19	429.2097	0.55	-	$[M+Na]^+$	451.2181	0.55	179.0560 $[M-C_{13}H_{22}O_3]^-$	$[M+COOH]^-$	$C_{19}H_{34}O_9$	√	√	√	√
15*	Geniposide	13.52	411.1262	-0.02	227.0926 $[M-C_6H_{10}O_5]^+$<br>209.0802 $[M-C_6H_{12}O_6]^+$	$[M+Na]^+$	-	-0.02	-	-	$C_{17}H_{24}O_{10}$	√	√	√	√
16	Rehmapicroside	13.7	369.150	0.02	167.1066 $[M-C_6H_{10}O_6]^+$	$[M+Na]^+$	345.155	0.02	165.0920 $[M-C_6H_{12}O_6]^-$	$[M-H]^-$	$C_{16}H_{26}O_8$	√	√	-	-

		9	9		149.0960 $[M-C_6H_{12}O_6-H_2O]^+$		8								

Table S1. Cont

No.	chemical name	RT/min	m/z ESI+	Error (ppm)	MS/MS	M+	m/z ESI--	Error (ppm)	MS/MS	M-	Formula	FRR	RRR	PRR	NRR
17	Echinacoside	15.14	804.2924	-0.35	625.2131 $[M-C_6H_{10}O_5]^+$ 479.1541 $[M-C_6H_{10}O_5-C_6H_{10}O_4]^+$	$[M+NH_4]^+$	785.2516	-0.35	-	$[M-H]^-$	$C_{35}H_{46}O_{20}$	√	√	√	√
18	Rehmaionoside A	15.59	413.2149	0.53	211.1693 $[M-C_6H_{12}O_6]^+$ 193.1584 $[M-C_6H_{12}O_6-H_2O]^+$	$[M+Na]^+$	435.2234	0.53	-	$[M+COOH]^-$	$C_{19}H_{34}O_8$	√	√	√	√
19	Cistanoside A	16.35	818.3079	-0.17	639.2275 $[M-C_6H_{10}O_5]^+$ 493.1702 $[M-C_6H_{10}O_5-C_6H_{10}O_4]^+$	$[M+NH_4]^+$	799.2676	-0.17	-	$[M-H]^-$	$C_{36}H_{48}O_{20}$	√	√	√	√
20	Rehmaionoside B	16.8	413.2146	0.05	211.1688 $[M-C_6H_{12}O_6]^+$ 193.1587 $[M-C_6H_{12}O_6-H_2O]^+$	$[M+Na]^+$	435.2231	0.05	-	$[M+COOH]^-$	$C_{19}H_{34}O_8$	√	√	√	√
21*	Acteoside	17.94	642.2389	-0.56	479.1545 $[M-C_6H_{10}O_3]^+$ 325.0919 $[M-C_6H_{10}O_3-C_8H_{10}O_3]^+$	$[M+NH_4]^+$	623.1979	-0.56	461.2390 $[M-C_6H_{10}O_5]^-$	$[M-H]^-$	$C_{29}H_{36}O_{15}$	√	√	√	√
22	Jionoside B1/B2	18.11	832.3241	0.27	653.2427 $[M-C_6H_{10}O_5]^+$ 507.1871 $[M-C_6H_{10}O_5-C_6H_{10}O_4]^+$	$[M+NH_4]^+$	813.2818	0.27	637.2331 $[M-C_{10}H_8O_3]^-$ 473.1676 $[M-C_{10}H_8O_3-C_6H_{12}O_5]^-$	$[M-H]^-$	$C_{37}H_{50}O_{20}$	√	√	√	√

23	Isoacteoside	18.77	647.195	0.41	479.1545 $[M-C_6H_{10}O_3]^+$ 325.0919 $[M-C_6H_{10}O_3-C_8H_{10}O_3]^+$	$[M+Na]^+$	623.198	0.41	461.2390 $[M-C_6H_{10}O_5]^-$	$[M-H]^-$	$C_{29}H_{36}O_{15}$	√	√	√	√

Table S1. Cont

No.	chemical name	RT/ min	m/z ESI+	Error (ppm)	MS/MS	M+	m/z ESI--	Error (ppm)	MS/MS	M-	Formula	FRR	RRR	PRR	NRR
24	Leucosceptoside A	19.65	661.2102	-0.36	485.1654 $[M-C_8H_{10}O_3]^+$ 339.1071 $[M-C_8H_{10}O_3-C_6H_{10}O_4]^+$	$[M+Na]^+$	637.2134	-0.36	461.1655 $[M-C_{10}H_8O_3]^-$ 473.1676 $[M-C_{10}H_8O_3-C_6H_{10}O4]^-$	$[M-H]^-$	$C_{30}H_{38}O_{15}$	√	√	√	√
25	Martynoside	21.86	675.2258	-0.29	-	$[M+Na]^+$	651.2293	-0.29	475.1788 $[M-C_{10}H_8O_3]^-$ 149.0622 $[M-C_{10}H_8O_3-C_{12}H_{22}O_{10}]^-$	$[M-H]^-$	$C_{31}H_{40}O_{15}$	√	√	√	√
26*	Fructose	2.275	-	0.6	-	-	179.0562	0.6	161.0456 $[M-H_2O]^-$ 113.0246 $[M-CH_6O_3]^-$	$[M-H]^-$	$C_6H_{12}O_6$	√	√	√	√
27*	D-(+)-Glucose	2.68	-	0.6	-	-	179.0562	0.6	161.0455 $[M-H_2O]^-$	$[M-H]^-$	$C_6H_{12}O_6$	√	√	√	√
28*	sucrose	3.87	-	0.02	-	-	341.109	0.02	179.0562 $[M-C_6H_{10}O_5]^-$ 161.0455 $[M-C_6H_{10}O_5-H_2O]^-$	$[M-H]^-$	$C_{12}H_{22}O_{11}$	√	√	√	-
29*	Melibiose	5.97	-	-2.21	-	-	341.1088	-2.21	179.0561 $[M-C_6H_{10}O_5]^-$ 161.0456 $[M-C_6H_{10}O_5-H_2O]^-$	$[M-H]^-$	$C_{12}H_{22}O_{11}$	√	√	√	√
30*	Raffinose	8.04	-	-0.18	-	-	503.161	-0.18	341.1089 $[M-C_6H_{10}O_5]^-$	$[M-H]^-$	$C_{18}H_{32}O_{1}$	√	√	√	√

Table S1. Cont

No.	chemical name	RT/min	m/z ESI+	Error (ppm)	MS/MS	M+	m/z ESI--	Error (ppm)	MS/MS	M-	Formula	FRR	RRR	PRR	NRR
							8		179.0562 $[M-C_6H_{10}O_5-C_6H_{10}O_5]^-$		6				
31*	Maltotriose	8.51	-	0.14	-	-	503.1616	0.14	341.1090 $[M-C_6H_{10}O_5]^-$ 179.0562 $[M-C_6H_{10}O_5-C_6H_{10}O_5]^-$	$[M-H]^-$	$C_{18}H_{32}O_{16}$	√	√	√	√
32*	Manninotriose	13.33	-	-0.26	-	-	503.1615	-0.26	341.1088 $[M-C_6H_{10}O_5]^-$ 179.0562 $[M-C_6H_{10}O_5-C_6H_{10}O_5]^-$	$[M-H]^-$	$C_{18}H_{32}O_{16}$	√	√	√	√
33*	Stachyose	17.73	-	0.67	-	-	665.2151	0.67	503.1616 $[M-C_6H_{10}O_5]^-$ 341.1090 $[M-C_6H_{10}O_5-C_6H_{10}O_5]^-$	$[M-H]^-$	$C_{24}H_{42}O_{21}$	√	√	√	-

Note: * indicates reference compound, √ indicates the presence of this compound, - indicates not detected.
